# Haplotype-based Pangenomics: A Blueprint for Climate Adaptation in Plants

**DOI:** 10.1093/gpbjnl/qzaf023

**Published:** 2025-03-10

**Authors:** Wanfei Liu, Peng Cui

**Affiliations:** Shenzhen Branch, Guangdong Laboratory for Lingnan Modern Agriculture, Genome Analysis Laboratory of the Ministry of Agriculture and Rural Affairs, Agricultural Genomics Institute at Shenzhen, Chinese Academy of Agricultural Sciences, Shenzhen 518120, China; Shenzhen Branch, Guangdong Laboratory for Lingnan Modern Agriculture, Genome Analysis Laboratory of the Ministry of Agriculture and Rural Affairs, Agricultural Genomics Institute at Shenzhen, Chinese Academy of Agricultural Sciences, Shenzhen 518120, China

Escalating impacts of climate change pose serious threats to ecosystems and agriculture, and thus to human societies and their living conditions. Plants — both wild and cultivated — are constantly facing global warming, drought, and precipitation change, rendering plant adaptability a crucial process. Haplotype-based pangenomics is a collection of genomes from each haplotype in multiple strains of a species, offering a cutting-edge approach for identifying traits that foster climate resilience. Although pangenomics focuses on building a pangenome for an agricultural crop, often within cultivars of a plant species, this technology holds significant untapped potential for preserving biodiversity, stabilizing ecosystems, and sequestering carbon.

Pangenomics has already revolutionized crop breeding, with major breakthroughs achieved in rice, maize, wheat, soybean, tomato, and potato [1], just to name a few. These pangenomic studies have been primarily focusing on crop breeding, adaptation, and evolutionary history [2]. In the context of climate-associated variables, developing a pangenome for non-agricultural species, such as forest trees, wetland plants, and grassland species, is also crucial for water regulation, soil stability, carbon storage, and biodiversity conservation. For instance, forest species such as oak (*Quercus*) and poplar (*Populus*) absorb significant amounts of CO_2_, acting as natural carbon sinks; wetland species such as *Spartina alterniflora* help prevent floods and store carbon in waterlogged soils; and grassland species in savannas and temperate grasslands have deep-rooted systems that help stabilize soils and store carbon, thereby preventing soil erosion and enhancing soil carbon. Applying pangenomics to these species helps uncover structural variants (SVs) associated with key traits, such as species adaptation and resilience to environmental changes [3,4]. The sexual reproduction genome always originates from a combination of two parental genomes, which may retain a highly heterozygous state. To solve the problem of high heterozygosity, a common strategy is to assemble two parental haplotypes into a chimeric genome. However, this strategy corrodes genomic variations between parents, which may be critical for the hereditability of diseases and phenotypes, and allele-specific gene expression [5,6]. This underscores the need for haplotype-based pangenomics and comparative genomics; the latter emphasizes cross-lineage genomic studies for major crops, such as those among the popular Poaceae and Solanaceae species.

Haplotype-based pangenomics presents a promising approach for analyzing environmental adaptability, as it can capture specific combinations of genetic variations that are crucial for resilience to changing conditions. Previous studies have demonstrated the role of haplotypes in plant climate adaptation and fruit quality heterosis [4,6]. In some highly heterozygous plants, such as trees that are closely related to ecosystems and climate, incorporating haplotypes facilitates the identification of key genes related to environmental adaptation.

A recent study led by Hansheng Zhao built a haplotype-based pangenome from 16 representative moso bamboo (*Phyllostachys edulis*) accessions (RMAs), providing valuable insights into genetic diversity, genomic architecture, climate adaptation, and population risks under future climate conditions [7]. This is the first pangenome of moso bamboo at the haplotype level, thus providing extensive genomic resources for future bamboo research.

One of the key findings is the extensive genetic variations between haplotypes rather than between accessions (97.0% *vs.* 3%), which assumed that there has been a significant difference between the two ancestral haplotypes in the common ancestor of moso bamboo populations. Among these RMAs, most inter-haplotype variations (68.5% and 68.8% for short variations and SVs, respectively) are detected across all accessions, while only a few inter-accession variations are present in all accessions (0.2% and 0.9% for short variations and SVs, respectively). Further analysis reveals that the number of single nucleotide polymorphisms (SNPs) among the same haplotypes is significantly lower than that among different haplotypes (637808 SNPs *vs.* 2933471 SNPs on average) by comparison among haplotypes of the three well-phased genomes.

To systematically compare genes and alleles of the 32 haplotype assemblies, the authors categorize their gene sets into core, softcore, dispensable, and private groups by their presence across accessions (called gene frequency), or into double-allele, single-allele, and variable-allele groups based on allele composition. The 12 groups, resulting from the combination of gene frequency and allele composition, possess different characteristics in terms of gene structure, expression pattern, and functional features. The core and double-allele gene sets have greater gene length, cDNA length, CDS number, and CDS size than the private and single-allele gene sets. Moreover, the average gene expression status gradually decreases from core to private gene sets in all allele composition groups, and gradually decreases from double-allele to single-allele gene sets in all gene frequency groups except for the private gene sets. Furthermore, the core and single-allele gene sets, which represent genes present in all accessions but only in one haplotype assembly, are related to environmental adaptations, such as stress tolerance, disease resistance, and DNA damage repair. These adaptation-related genes are also very important and exploitable in other species.

Allele-specific expression gene sets have a high degree of accession specificity (81.8% in 1–2 accessions *vs.* 0.1% in all accessions), exhibit tissue-specific expression patterns (72% variable tissue expression patterns *vs.* 28% consistent tissue expression patterns), and are associated with environmental adaptations, such as stimuli and defense responses and tissue-specific developmental processes.

The genotype–environment association analysis identifies 1050 adaptive variations associated with bioclimatic variables, revealing a greater contribution of climate effects than that of geography (35% *vs.* 13% of adaptive variations). The risk of non-adaptedness analysis and gradient forest modeling indicate that the northern and western regions may have been facing adaptive risks under the high-emission scenario.

However, current genomic offset models — used to predict how species will respond to climate changes — are limited [8]. These models often fail to account for epigenetic factors (changes in gene activity not caused by changes in DNA sequences) and complex interactions between genes and environments. To overcome these barriers, future models must integrate multi-omics approaches (such as population genomics, transcriptomics, epigenetics, metabolomics, and microbiomics) and adjust to changing environmental conditions (both present and future).

In addition, the application of pangenomics to climate adaptation poses significant technical challenges. First, the large genomes of many plants make sequencing expensive and resource-intensive. Next, current pangenome methodologies largely rely on linear genome models, which pose challenges for integrating multiple genomes from the same species and ensuring accessibility of the integrated representation to biologists. Furthermore, many types of genomic resources, such as haplotype information critical for understanding climate adaptation, remain underutilized or are wasted in pangenome analyses due to technical limitations [7]. This emphasizes the need for the development of new analytical techniques and computational methods to more accurately represent and analyze pangenomic data.

Although promising, the application of pangenomics in climate adaptation poses several ethical and socioeconomic challenges. For example, intellectual property concerns exacerbate inequalities by restricting access to climate-resilient technologies in developing nations. Smallholder farmers in sub-Saharan Africa may struggle to afford drought-resistant seeds due to high costs and patent restrictions. The Nagoya Protocol, which aims to ensure fair benefit-sharing with indigenous communities, provides a framework for equitable access. However, more must be done to enhance fair benefit-sharing mechanisms and to ensure equitable technology distribution. Expanding global funding and supporting subsidized research will help bridge the gap between technological innovation and global food security [9].

Artificial Intelligence (AI) and Machine Learning (ML) are transforming genomic analyses by accelerating the discovery of complex traits required for climate adaptation. AI models are increasingly used to identify SVs and gene–environment interactions [8,10]. When integrated with haplotype-based pangenomes, AI and ML enhance the ability to discern intricate genetic patterns and predict phenotypic outcomes under varying environmental conditions [7]. Additionally, advanced techniques, such as HiFi sequencing, combined with SV analysis, improve the detection of large genomic changes, thereby deepening our understanding of adaptive traits. However, high costs remain a barrier, particularly for developing countries.

Modern-day genome editing tools, such as CRISPR-Cas9, have been extensively explored to understand and develop stress-tolerant plant varieties to mitigate the negative effects of climate changes. CRISPR-Cas9 has frequently been employed to understand the stress-responsive genes of heat, drought, salt, and heavy metals, which can be used to enhance climate resilience [11]. Genome editing tools also provide solutions and strategies for plant conservation or restoration, although there are challenges, such as obtaining efficient transformants, producing transgene-free plants, achieving homozygous mutants, and assessing ecological risks [12].

The success of the pangenome depends on global collaboration, open-access data, and continued integration of AI and gene-editing technologies. Projects such as the Earth BioGenome Project and the Consultative Group on International Agricultural Research are already promoting cross-border sharing of genomic data, allowing researchers to apply insights where they are most needed. Reducing costs and expanding data-sharing efforts are essential to ensure that AI-enhanced genomic tools can be effectively leveraged for global climate resilience.

Looking forward, the convergence of long-read sequencing, AI, and CRISPR is set to revolutionize agriculture and biodiversity conservation. Pangenomics based on long-read sequencing can identify genomic variations related to key traits, AI-based genomic analyses can uncover genomic variations associated with agronomic traits and climate changes, and CRISPR-based genome editing tools can accelerate breeding improvements for environmental adaptation. By fostering global cooperation and making genomic technologies accessible, the scientific community can help create climate-resilient species and protect critical ecosystems.

In conclusion, haplotype-based pangenomes are a promising solution for climate adaptation challenges. By extending genomic tools beyond agriculture to include keystone species that are critical for carbon sequestration and ecosystem stability, climate resilience can be built across multiple fronts. Through global collaboration, integration of AI and precision breeding, and addressing ethical and socioeconomic barriers, we can ensure that genomic innovations benefit all regions. With sustained investments in data-driven research, plants — and the ecosystems they support — can be equipped to thrive in a rapidly changing world.

## CRediT author statement

**Wanfei Liu:** Writing – original draft, Writing – review & editing. **Peng Cui:** Conceptualization, Funding acquisition, Writing – original draft, Writing – review & editing. Both authors have read and approved the final manuscript.

## Competing interests

Both authors have declared no competing interests.
